# Supplementary material for: Characterization of Pseudofusicoccum Species from Diseased Plantation-Grown Acacia mangium, Eucalyptus spp., and Pinus massoniana in Southern China
Source: Pathogens. 2023 Apr 8;12(4):574. doi: 10.3390/pathogens12040574 (PMC10142214; doi:10.3390/pathogens12040574)
Supplement: Supplementary file 1 [file pathogens-12-00574-s001.zip › pathogens-2298028-supplementary.pdf]

pathogens

MDPI

Supplementary Materials

Characterization of *Pseudofusicoccum* Species from Diseased Plantation-Grown *Acacia mangium*, *Eucalyptus* spp., and *Pinus massoniana* in Southern China

Guoqing Li, Wenxia Wu, Linqin Lu, Bingyin Chen and Shuaifei Chen*

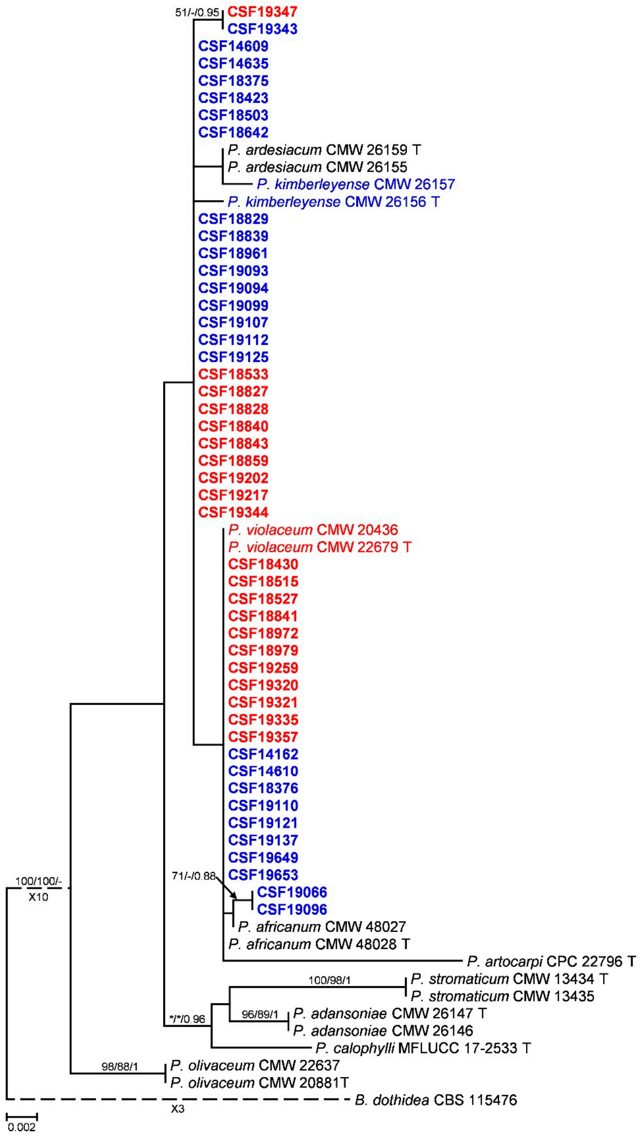

Figure S1. Phylogenetic tree based on maximum likelihood (ML) analyses of the ITS locus for *Pseudofusicoccum* species. Isolates in blue (*P. kimberleyense*) and red (*P. violaceum*) colors in bold were sequenced in this study. Bootstrap support values ≥ 70% for ML and MP (maximum parsimony) and probabilities values of BI (Bayesian inference) ≥ 0.9 are presented above the branches as follows: ML/MP/BI, bootstrap support values < 70% and probabilities values < 0.9 are marked with '*', and absent are marked with '-'. Ex-type isolates are marked with 'T'. The trees were rooted in *Botryosphaeria dothidea* (CBS 115476).

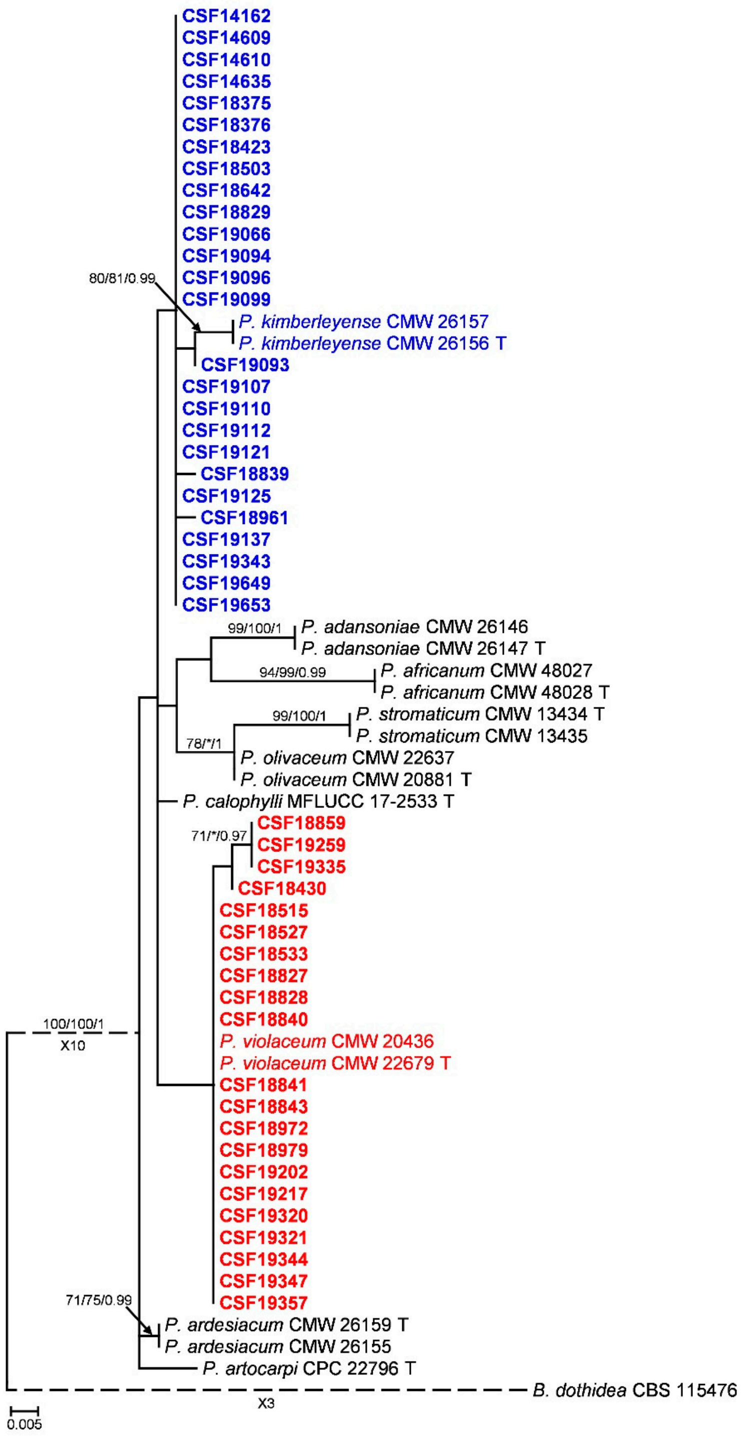

Figure S2. Phylogenetic tree based on maximum likelihood (ML) analyses the *tef1* locus for *Pseudofusicoccum* species. Isolates in blue (*P. kimberleyense*) and red (*P. violaceum*) colors in bold are sequenced in this study. Bootstrap support values ≥ 70% for ML and MP (maximum parsimony) and probabilities values of BI (Bayesian inference) ≥ 0.9 are presented above the branches as follows: ML/MP/BI, bootstrap support values < 70% and probabilities values < 0.9 are marked with '*', and absent are marked with '-'. Ex-type isolates are marked with 'T'. The trees were rooted in *Botryosphaeria dothidea* (CBS 115476).

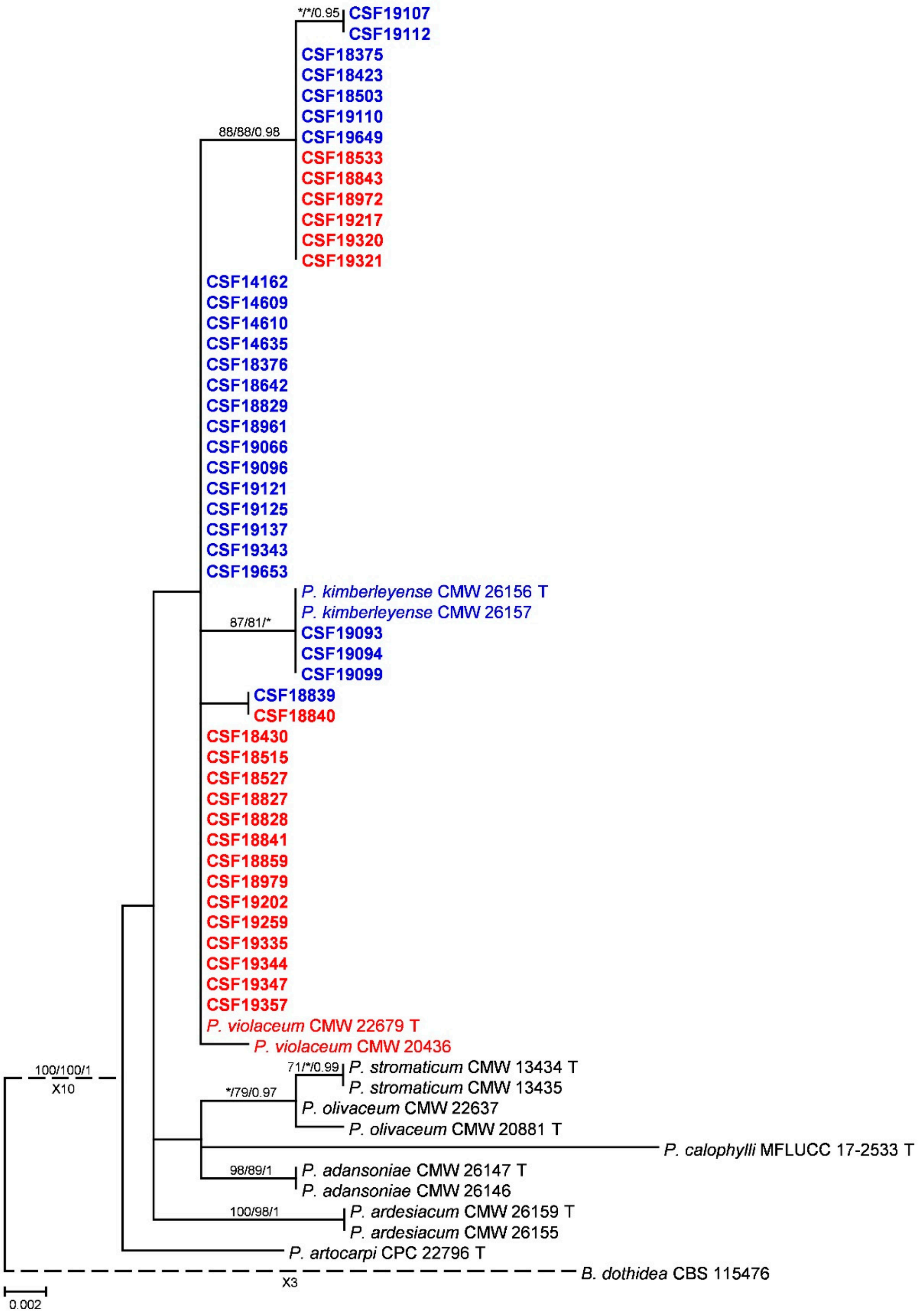

Figure S3. Phylogenetic tree based on maximum likelihood (ML) analyses the *tub2* locus for *Pseudofusicoccum* species. Isolates in blue (*P. kimberleyense*) and red (*P. violaceum*) colors in bold are sequenced in this study. Bootstrap support values ≥ 70% for ML and MP (maximum parsimony) and probabilities values of BI (Bayesian inference) ≥ 0.9 are presented above the branches as follows: ML/MP/BI, bootstrap support values < 70% and probabilities values < 0.9 are marked with '*', and absent are marked with '-'. Ex-type isolates are marked with 'T'. The trees were rooted in *Botryosphaeria dothidea* (CBS 115476).
